# Retinal vasculitis and ocular vitreous metastasis following complete response to PD-1 inhibition in a patient with metastatic cutaneous melanoma

**DOI:** 10.1186/s40425-014-0041-1

**Published:** 2014-12-16

**Authors:** Joshua S Manusow, Leila Khoja, Nataly Pesin, Anthony M Joshua, Efrem D Mandelcorn

**Affiliations:** University of Toronto Department of Ophthalmology and Vision Sciences, University Health Network/Toronto Western Hospital, 399 Bathurst Street, 6 East Room 415, Toronto, Ontario M5T 2S8 Canada; Department of Medical Oncology, Princess Margaret Cancer Centre, Toronto, Canada

**Keywords:** Vitreous metastasis, Melanoma, Retinal vasculitis, PD-1 inhibitor, Melanoma-associated Retinopathy (MAR)

## Abstract

We report on a 36-year-old woman treated with the anti PD-1 antibody Pembrolizumab for metastatic cutaneous melanoma in the first line setting. She achieved a complete response and then relapsed with metastases to the vitreous cavity with an associated angiographically determined retinal vasculitis. Vitreous metastasis without choroidal involvement is unusual and may be due to individual cell extravasation, vitreous hemorrhage containing malignant cells, or direct spread through the optic nerve. This finding highlights the need for immune sanctuary sites to be monitored in the presence of PD-1 inhibition and we hypothesize that the use of PD-1 inhibitor potentiated the patient’s angiographically determined retinal vasculitis.

## Background

Pembrolizumab is an anti-programmed cell death-1 antibody (PD-1) currently under clinical evaluation primarily for metastatic cutaneous melanoma as well as lung cancer. The activity and relapse characteristics of melanoma and pembrolizumab in immune sanctuary sites has been incompletely reported to date. In this case report, we identify a 36 year old female who achieved a complete response and then relapsed with angiographically determined retinal vasculitis and melanoma cells within the vitreous cavity. Isolated vitreous metastasis is rare with just 18 previously reported cases in the world literature [1] and highlights the issue of melanoma within immune sanctuary sites in the setting of PD1 inhibition. We discuss the possible mechanisms and diagnostic pitfalls associated with this condition.

## Case presentation

A 36-year-old female, previously well, presented with a 2.4 mm ulcerated melanoma, with 1 mitosis/10 high powered fields, over her left back. The remainder of her medical, family, and social history was noncontributory. Following initial wide local resection and negative sentinel lymph node biopsy, she remained well with normal surveillance CT scans. Five years later, she developed hematuria and was found to have melanoma bladder metastases. Whole body staging investigations revealed a normal MRI brain and widespread metastatic disease in the perinephric soft tissue, left adnexa, lung and pleura. BRAF mutation testing of her primary was negative and she was enrolled into a phase I clinical trial of pembrolizumab (10 mg/kg q3 weekly) approximately 5 months after the diagnosis of metastatic disease. After 3 doses, she complained of left sided blurred vision and mild anterior uveitis was diagnosed which was treated with prednisolone eye drops. She continued on pembrolizumab and was documented to be in complete response after 15 treatments (approximately 10 months of treatment). Shortly thereafter, she was admitted with grade 3 pneumonitis secondary to pembrolizumab treatment, requiring intravenous steroids. A full body CT scan confirmed ongoing complete response. At this time she also complained of blurred vision.

**Figure 1:**
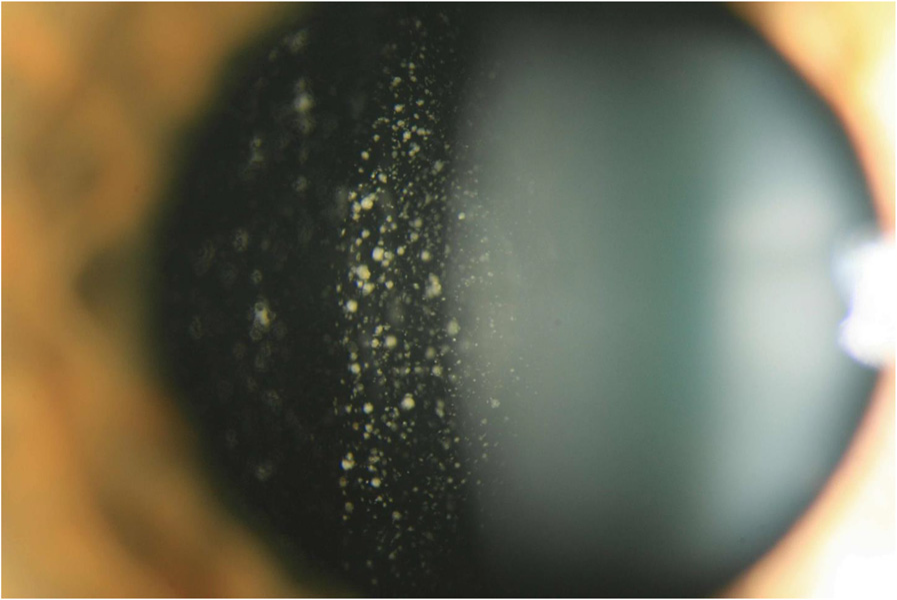
**Retrolental cells.** Slit lamp photograph of the left eye disclosing cohesive, pigmented, retrolental cells.

**Figure 2:**
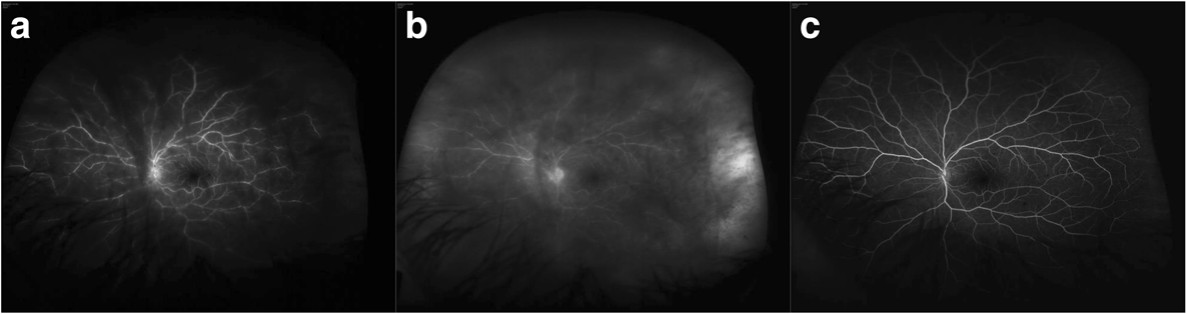
**Retinal vasculitis.** Fluorescein angiogram of the left eye discloses **a)** diffuse retinal vasculitis in the mid frame with **b)** leakage in the late frame and **c)** resolution of vasculitis following vitrectomy.

On examination her visual acuity was 20/20 OD and 20/200 OS. Her intraocular pressures were normal. Slit-lamp examination of both eyes was unremarkable. Her left fundus examination demonstrated clumped cellular material in the vitreous [Figure 1], more so in the anterior than posterior vitreous. The vitreous cells did not have the classical clinical appearance of inflammatory cells, as they were adherent in small clusters with no collateral destruction of the surrounding vitreous gel. Fluorescein angiography (IVFA) [Figure 2a-b] demonstrated significant angiographically determined retinal vasculitis with leakage from retinal vessels. There was no macular edema. Laboratory testing for syphilis and toxoplasmosis was negative. A MRI did not appear to show brain metastasis.

With high suspicion for metastatic melanoma to the vitreous, a diagnostic vitrectomy was performed.

**Figure 3:**
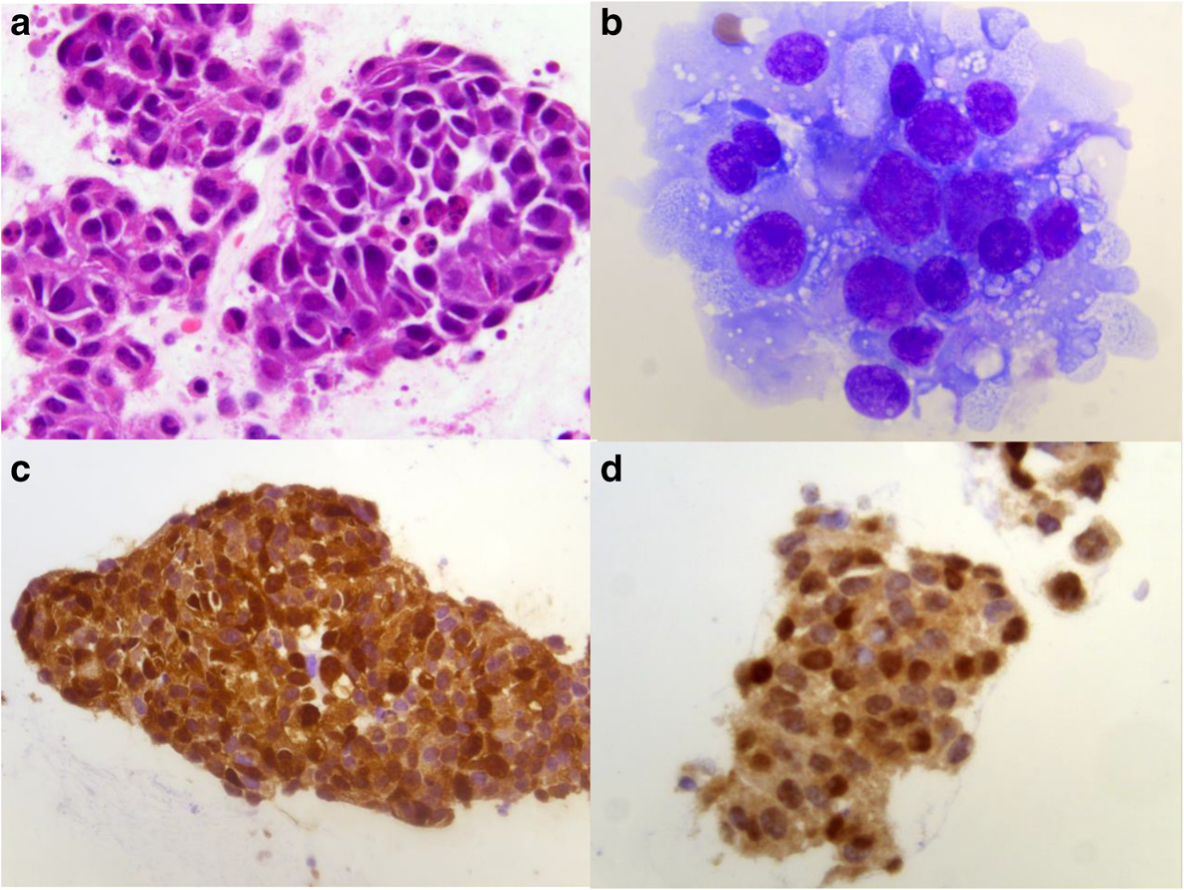
**Histopathology.** Photomicrograph shows cohesive groups of malignant epithelioid cells displaying pleomorphic round eccentric nuclei with prominent nucleoli and abundant cytoplasm. **a)** Hematoxylin and eosin, 63× **b)** Diff-Quik, 100× . Immunohistochemistry discloses positive staining for **c)** S-100 stain, 40× and **d)** microphthalmia stain, 63×.

The vitreous biopsy contained tightly cohesive groups and isolated tumour cells with pleomorphic nuclei and prominent nucleoli [Figure 3]. The cells stained positively for vimentin, S-100 protein, Melan A, HMB45, and pancytokeratin (AE1/AE3) by immunohistochemistry. These findings confirmed the diagnosis of metastatic malignant melanoma in the vitreous cavity.

The patient improved post-operatively with a visual acuity of 20/20. A repeat IVFA demonstrated complete resolution of retinal vascular leakage (Figure 2c) and a 24–2 humphrey visual field was entirely normal. A post-operative electroretinogram (ERG) demonstrated normal rod and cone function and no flat B-wave.

Two months later the patient presented with acute onset apraxia but no other neurological deficits. A 7 mm post-gadolinium enhancing nodule associated with intraparenchymal hematoma suggestive of brain metastasis was diagnosed by MRI. The previous MRI was reviewed and it was determined that this nodule was present at a size of 1 mm on the initial scan pre-vitrectomy. Shortly thereafter, she had a recurrence of refractile retrolental cells in the left eye and a few vitreous cells in the right eye and was subsequently treated with external beam radiation (20 Gy). She is currently being actively followed with surveillance imaging. The lesion in the brain subsequently resolved spontaneously on MRI and she remains in radiological complete response.

## Conclusions

Though cutaneous melanoma commonly metastasizes to the lymphatic system, central nervous system, liver, subcutaneous tissues, and lung, it accounts for only 2 % of primary tumors metastasizing to the uvea. [2] Our case highlights the unusual nature of metastases directly to the vitreous humor with no evidence of uveal involvement. The mechanism of metastases to the vitreous is unclear but may include (i) migration/extravasation of single malignant cells through the retinal vessels into the retrohyaloidal space [1] (ii) migration with vitreous hemorrhage, originating from retinal vessels containing malignant cells [1] or (iii) seeding directly through the optic nerve [2]. In our case, despite the initial report of a negative MRI, a brain nodule did exist making the mechanism of optic nerve seeding possible. A single intraretinal hemorrhage noted intra-operatively may have also been the point of access of the malignant cells [1].

The development of secondary disease within the eye despite a radiological complete response highlights the phenomenon of recurrence within immune privileged sites. These are classically considered to be the eyes, placenta or testes but not the brain or central nervous system (CNS) per se [3]. Such sites lack or have minimal lymphatic drainage and have an immunosuppressive environment thought to be protective under normal physiological circumstances. The CNS is only partially protected against the systemic immune response by the blood brain barrier as it has resident macrophages. Dendritic cells necessary for antigen presentation and T-cell activation are additionally able to circulate in the meninges and choroid plexus but not in the normal parenchymal tissue or perivascular spaces. Therefore even with highly active drugs such as targeted therapy in the BRAF mutant population or with the new checkpoint inhibitors (CTLA-4 and PD-1/PDL-1 inhibitors) which potentiate the immune response in tumor, relapse at immune privileged sites will continue to be problematic. Ipilimumab, a CTLA-4 inhibitor, has shown efficacy in the treatment of brain metastases in a small phase II study [4]. Trials to date on Pembrolizumab have allowed recruitment of patients with stable brain metastases (not requiring steroid treatment) but specific activity in the CNS is yet to be reported [5]. To our knowledge, there are no reports of vitreous relapse with ipilimumab.

Additionally, this patient presented with significant angiographically determined retinal vasculitis. This vasculitis may be related directly to the ocular metastasis or a paraneoplastic phenomenon. The fact that the angiographically determined vasculitis completely resolved immediately following vitrectomy (Figure 2c) in the absence of any other treatment suggests that it is a local immune/paraneoplastic effect. Paraneoplatic associated phenomena are reported in the retina, including melanoma associated retinopathy (MAR) and cancer associated retinatopathy (CAR). These present with photopsia, loss of vision, negative electroretinographic waveforms, central scotoma, retinal vessel attenuation, vasculitis, vitritis, macular edema and eventually retinal atrophy [6,7]. The common mechanism is molecular mimicry; malignant cells that are antigenically similar to retinal antigens stimulate the immune system and lead to autoimmune injury of the retina. If our patient’s vasculitis is related to a paraneoplastic phenomenon, the lack of electronegative ERG suggests that a non-bipolar cell retinal antigen was targeted. The physiological role of the PD-1/PDL1 axis is believed to protect tissue from immune recognition. Importantly, limited evidence suggests that it may also protect the retinal pigment epithelia (RPE) which may explain the absence of RPE and ERG changes in our patient [8].

One hypothesis is that the PD-1 inhibitor potentiated an immune response against either similar retinal antigens or melanoma cells within the retinal vessels leading to inflammation of surrounding peripheral tissue and secondary angiographically determined retinal vasculitis in our patient. Indeed, reported toxicities from this class of drugs include other autoimmune/inflammatory events including nephritis, thyroiditis and endophthalmitis [9]. Thus, we suspect that the angiographically determined retinal vasculitis in our case may have been a paraneoplastic phenomenon to non-bipolar cell retinal antigens epitopically similar to those of melanoma. Alternatively, the angiographically determined retinal vasculitis could have been the result of an enhanced, local, inflammatory response to melanoma cells in an *anti*-PD-1 molecular environment which showed dramatic resolution once the melanoma cell burden was removed by vitrectomy.

In summary, we present the rare case of cutaneous melanoma with metastasis to the vitreous cavity without choroidal involvement but associated angiographically determined retinal vasculitis in the presence of PD1 blockade with pembrolizumab. This case highlights the need for vigilance in dealing with check-point blockade antibodies and immune privileged sites, as well as the need to be aware of a broad range of auto-immune phenomena that can occur.

## Consent

Written informed consent was obtained from the patient for publication of this Case report and any accompanying images. A copy of the written consent is available for review by the Editor-in-Chief of this journal.

## Abbreviations

PD-1: Programmed cell death-1
OD: Right eye
OS: Left eye
ERG: Electroretinogram
CNS: Central nervous system
MAR: Melanoma associated retinopathy
CAR: Cancer associated retinopathy
RPE: Retinal pigment epithelia
IVFA: Intravenous Fluorescein Angiogram
